# Young professionals for health development: the Kenyan experience in combating non-communicable diseases

**DOI:** 10.3402/gha.v6i0.22461

**Published:** 2013-11-20

**Authors:** Duncan M. Matheka, Joseph Nderitu, Rajesh Vedanthan, Alessandro R. Demaio, Mellany Murgor, Kiti Kajana, Poonamjeet Loyal, Faraj O. Alkizim, Sandeep P. Kishore

**Affiliations:** 1 Department of Medical Physiology, School of Medicine, University of Nairobi, Nairobi, Kenya; 2 Young Professionals Chronic Disease Network, Boston, MA, USA; 3 Department of Human Anatomy, School of Medicine, University of Nairobi, Nairobi, Kenya; 4 Zena and Michael A. Wiener Cardiovascular Institute, Icahn School of Medicine at Mount Sinai, Mount Sinai Medical Center, New York, NY, USA; 5 Department of International Health, Immunology and Microbiology, Copenhagen School of Global Health, University of Copenhagen, Copenhagen, Denmark; 6 Harvard Global Equity Initiative, Department of Global Health and Social Medicine, Harvard Medical School, Boston, MA, USA; 7 Global Health Strategies, New York, NY, USA; 8 Weill Cornell/The Rockefeller University/Sloan-Kettering Institute, New York, NY, USA

**Keywords:** diabetes mellitus, cancer, cardiovascular diseases (CVD), rheumatic heart disease (RHD), youth, young people, medical students, developing countries, Africa, screening medical camps

## Abstract

Young individuals (below 35 years) comprise an estimated 60% of the global population. Not only are these individuals currently experiencing chronic, non-communicable diseases (NCDs), either living with or at risk for these conditions, but will also experience the long-term repercussions of the current NCD policy implementations. It is thus imperative that they meaningfully contribute to the global discourse and responses for NCDs at the local level. Here, we profile one example of meaningful engagement: the Young Professionals Chronic Disease Network (YPCDN). The YPCDN is a global online network that provides a platform for young professionals to deliberate new and innovative methods of approaching the NCD challenges facing our societies. We provide a case study of the 2-year experiences of a country chapter (Kenya) of the YPCDN to demonstrate the significance and impact of emerging leaders in addressing the new global health agenda of the 21st century.

Non-communicable diseases (or NCDs, including diabetes mellitus, cardiovascular diseases [CVD], cancers and chronic respiratory diseases) are now the leading causes of death globally. They account for more than 35 million of the 53 million annual deaths worldwide (1–3). Of these, more than 75% occur in low- and middle-income countries (LMICs) (1, 2). In Kenya, it is estimated that over 50% of all hospital admissions and 55% of hospital deaths are due to NCDs (4, 5).

In LMICs, NCDs are more prevalent among youth as compared to high-income countries (HICs) where elderly populations exhibit greater prevalence of NCDs (6, 7). In addition, classic risk behaviors for NCDs such as smoking, alcohol abuse, physical inactivity and unhealthy diets are common among the youth in LMICs. In Kenya, an estimated 32.2% of school-going children and 53% of college students smoke (6–8).

According to demographic studies, young people (<35 years of age) comprise the majority (up to 60%) of the world's population (9, 10). They are accustomed to modern technologies, and offer innovative ideas and inspiring enthusiasm (11, 12). With proper empowerment, they can contribute enormously to combating NCDs (11).

## The Young Professionals Chronic Disease Network

Founded in 2009, the Young Professionals Chronic Disease Network (YPCDN) is a global network of early-career professionals committed to addressing NCDs. Driven by and for young people, YPCDN aims to connect and mobilize emerging young health leaders from all technical backgrounds – providing a platform for interaction, collaboration, support, and knowledge sharing. Through an online portal and regional group meetings, members drive policy analysis, local and global activism, engage in peer education, and contribute to social and academic discourse. YPCDN now has a collective membership of more than 2,000 members across 105 countries – within many of which, local chapters have been established.

## Youth-led initiatives to address NCDs in Kenya

Medical students at the University of Nairobi launched the Kenyan chapter of YPCDN in September 2011, concurrent with the United Nations High-level Meeting on NCDs (13). Since then, the chapter has grown to accommodate all five public medical schools in the country and offers young professionals the opportunity to discuss and formulate solutions to the growing and already enormous NCD burden in Kenya. The Kenyan YPCDN chapter undertakes a number of core tasks and activities.

### Leadership and global engagement

Local chapters meet every third Thursday of the month (dubbed 3T project) to discuss NCDs, and their reports are shared within YPCDN's global online platform (14, 15). This allows them to network and collaborate with other similar groups. These discussions have the effect of generating and sustaining interest and enthusiasm among young professionals (Table 1).

**Table 1:** List of activities carried out by the Kenyan chapter of YPCDN within a 2-year period (Sept 2011–Sept 2013)

Activity	Description
Peer leadership and mentorship	Groups are led by young people, under the mentorship of seniors in the global health field
3T meetings and networking	Meetings held every Third Thursday (3T) provide opportunities to plan and network locally/globally
Global engagement	Global partnerships have been in research, 3Ts, NCDFREE project and UN ECOSOC meetings
Research and publications	Young people have been involved in collaborative research and publications on NCDs
Advocacy	Group is active in advocacy: World No Tobacco Day (WNTD) and NCDFREE Project (15)
Community screening camps	Screening camps for DM/HTN and creating awareness on cancer and healthy lifestyles to prevent NCDs
Heath education	Talks on RHD and healthy lifestyles have been held in schools, churches and markets
Local partnerships	There have been local partnerships with NGOs, Ministry of health, schools, churches, hospitals

DM = diabetes mellitus; ECOSOC = United Nations Economic and Social Council; HTN = hypertension; NCDs = non-communicable diseases; NGOs = non-governmental organizations; RHD = rheumatic heart disease; Sept = September; UN = United Nations; YPCDN = Young professionals Chronic Disease Network.

### Research and advocacy

Through the aforementioned 3T meetings, the discussions often stimulate research and advocacy (Table 1). Major progress has been made in this regard despite numerous challenges. The Kenyan chapter is participating in the YPCDN'S NCDFREE project, which aims to build a social and political voice for NCDs through the creation of a series of films focusing on inspiring change makers from developing countries including Kenya, Ghana and Mongolia (15). This would encourage stakeholders and young people all over the world to take more interest in their local settings (15).

Tackling NCDs requires multidisciplinary input from the government, education, health, industrial, civil society, and private sectors. The Kenyan chapter is currently advocating for collective efforts in the fight against NCDs by working with several organizations including Kenya's Ministry of Health – Division of NCDs. Focus has been on tobacco control, cancer, diabetes mellitus and hypertension screening, and rheumatic heart disease (RHD) awareness. With the media, it aims to enlighten the youth on behavioral changes that can reduce their risk of being affected by NCDs. There are however many hurdles in advocacy, especially in countering the role of tobacco industries in the NCD problem (14).

Through YPCDN, Kenyan young professionals have had opportunities to connect with other emerging health leaders elsewhere, and even collaborate in research and publication. Besides the current manuscript, other papers written in collaboration include: (1) Shining a Light on world's biggest killer: non-communicable diseases; (2) Pattern, knowledge and practices of HbA_1C_ testing among diabetic patients in a Kenyan tertiary referral hospital; (3) Complementary and alternative medicine use among diabetic patients in Africa: a Kenyan perspective; (4) Unregulated complementary and alternative medicine use among diabetic patients in Africa: A call for action; (5) NCDs and Africa: a forgotten burden and a barrier to development; among others (16).

Through this network, Kenyan youth delegates have also been involved in high-profile national and international decision-making meetings such as: the 2012 New York City United Nations Economic and Social Council (ECOSOC) Round-table Meeting on ‘NCDs in Children and Adolescents’, and the 2013 Geneva ECOSOC Meeting on ‘Young people, NCDs and Technology’ (17). Moreover, the chapter was represented in the 2013 Harvard launch of the NCDFREE project and the Harvard NCD Child seminar on ‘NCDs and Disability’ (15). In Kenya, the group has worked with the Ministry of Health and World Health Organization on the 2013 World No Tobacco Day (WNTD) as well as on setting up RHD patient support clubs in Kenya (15, 17).

### Community screening programs

The rising prevalence of NCDs and infectious diseases in LMICs such as Kenya leads to a double burden of disease, and medical care is not readily accessible to the majority of the citizens (1, 2, 9, 18). Therefore, free screening medical camps are a useful way to reach those in need of medical services from these poor communities. They provide an avenue where screening, early diagnosis, and early linkage to healthcare facilities can be made so as to curb progression of diseases to potentially non-reversible complications. The Kenyan YPCDN chapter has championed local impact through conducting such free screening medical camps every month (Table 1).

**Fig. 1:**
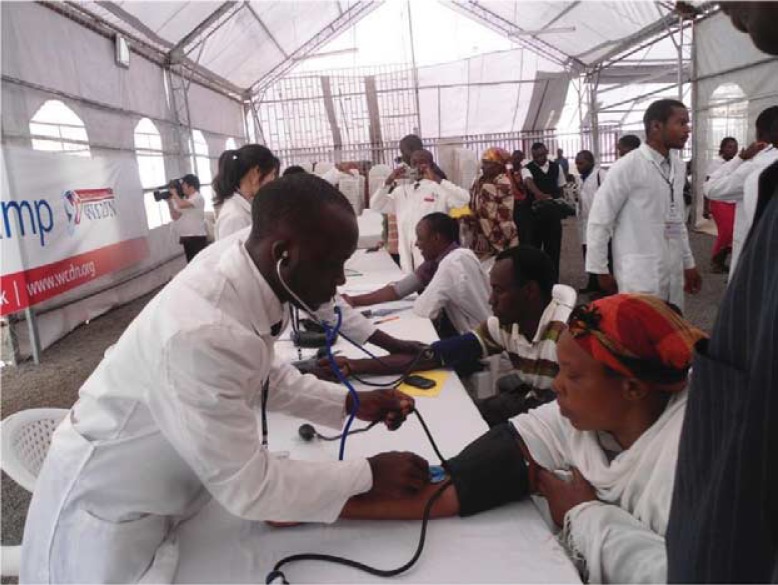
Medical students taking blood pressure measurements during a medical camp in Nairobi, Kenya.

The first screening camp organized by the Kenyan YPCDN chapter, held on 19th September 2011, screened 803 members of the public for diabetes mellitus and hypertension. In addition, individuals were educated on these diseases and those diagnosed with diabetes or hypertension referred to healthcare facilities for management and follow-up. The group has held several other screening camps in various parts of the country. These camps are held in partnership with other organizations – NGOs, churches, schools, and hospitals (Fig. 1, Table 1).

**Fig. 2:**
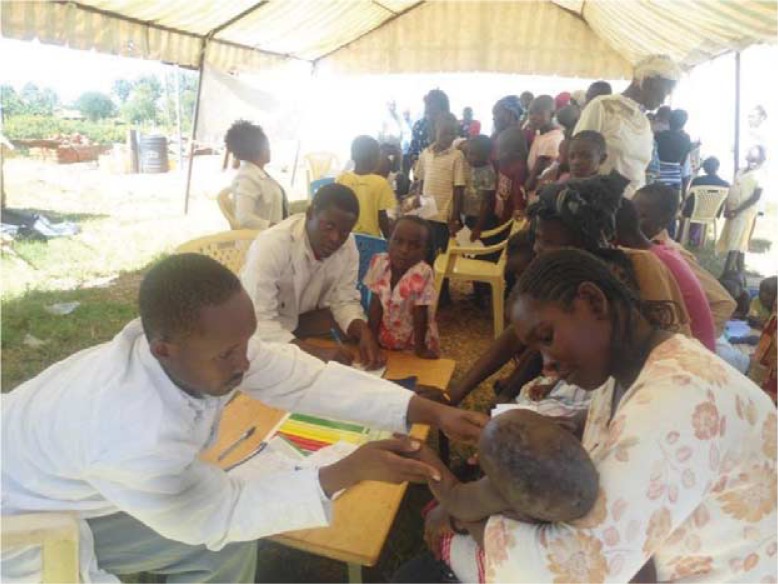
Young professionals attending to patients during a medical camp in Siaya, Kenya.

Medical camps provide experiential training for medical students, thus complementing their academic and clinical experiences. The students are exposed to public health realities and the challenges faced by individuals while addressing their health concerns (Fig. 2). Such experiences provide a rich learning environment for the medical students that better prepare them for their medical career (19).

### Peer education and youth engagement

**Fig. 3:**
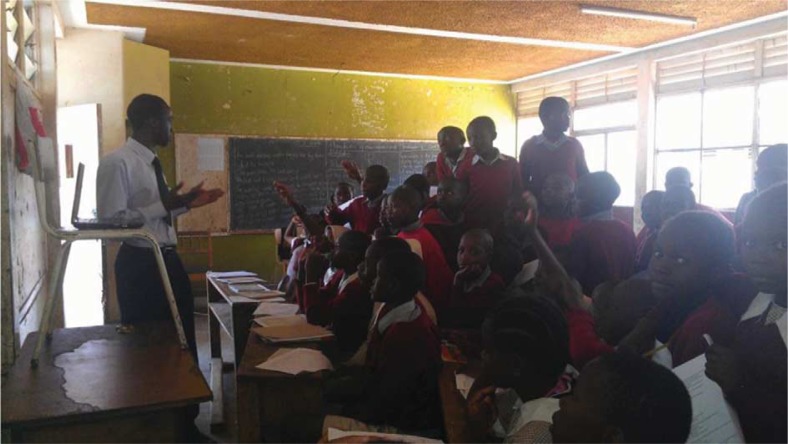
Medical student conducting NCD Training at Toi Primary School, Nairobi, Kenya.

Creating awareness on NCD risk factors, prevention, and early management are essential elements in the mitigation of NCDs. Bearing this in mind, YPCDN has provided presentations in primary and secondary schools around Kenya to teach children how to prevent NCDs as well as ways to ensure early detection (Fig. 3).

**Fig. 4:**
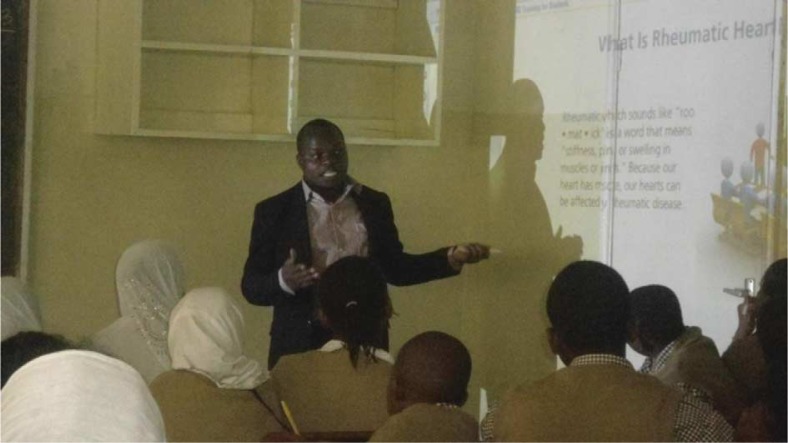
Young professional using an interactive module to conduct RHD training at Bellevue Primary School, Nairobi, Kenya.

In one of these projects, the training utilized innovative technology (an interactive digital module) to train school-going children on RHD (20). The module developed by WiRED international (a US-based non-profit organization working in Kenya) had simplified animated presentations linking sore throat, rheumatic fever, and RHD, as well as prevention strategies. The module also introduced questions throughout the presentation and provided instant feedback to reinforce key concepts (Fig. 4) (20).

By educating children at an early age, most will retain the message as they grow up, and also pass the message on to their families and peers during school holidays (21, 22). They may also adjust any high-risk diets and social habits as well as those of their families (21, 22). Notably, students in high schools are drawn from diverse backgrounds and geographical locations, and this could be a platform to reach even the communities in remote parts of the country (21, 22).

Teachers are also targeted during the training sessions so that they can act as reference points for the students who are in constant contact with them. The long-term aim is to have the inclusion of NCD education as part of school curricula, which is the case with some infectious diseases. The health education seminars have also been held during religious gatherings and in market areas.

## Discussion

In this case study, we highlighted the experiences of a country chapter (Kenya) of YPCDN to demonstrate the significance and impact of emerging leaders in addressing NCDs. Through this chapter, both clinical and educational programs have been implemented via mentorship, global engagement, meetings, networking, research and advocacy, community screening camps, health education programs, and local partnerships (Table 1).

The Kenyan chapter of YPCDN is mainly composed of and led by medical students at the University of Nairobi. Of the global YPCDN chapters, the Kenyan chapter is currently the most active in an LMIC setting. This is because of its catchment of a pool of energetic medical students, thus easy to have regular monthly meetings at the University of Nairobi (14). It is also the first in Africa, although there are plans to set up more groups in LMICs and hopefully offer a comparison on how various developing countries address NCDs (14, 15). Chapters in HICs (London, Geneva, Copenhagen, Boston, and New York) are mainly composed of young professionals in the working field and often from different organizations (14). These chapters in HICs provide mentorship, encouragement, and support to those in Kenya and other LMICs.

The Kenyan chapter of YPCDN is not a lone player and has embraced bidirectional partnerships. It has collaborated with other locally sponsored programs to address NCDs. In most cases, it has initiated its own programs and sought partners such as schools, churches, and hospitals. The group has also been invited to partner in projects led by hospitals, Ministry of health, NGOs, churches, and private companies. The group therefore complements activities by other stakeholders, and embraces multisectoral action in fighting NCDs (14, 15).

However, there are some challenges faced by the Kenyan chapter of YPCDN. For example, in the course of running the projects, the key challenge has been the lack of finances, given that most of the members are students who often have no income and mostly depend on external funding. In addition, emerging leaders and youth are often ignored or dismissed by senior policy makers and professionals. Despite their potential for positive change, young people often lack credentials and experience, and thus are often left out of policy making and governance discussions. With such obstacles, there is weakened morale and loss of membership.

## Conclusion

Emerging leaders and young professionals are active players in the global efforts to combat NCDs. Efforts by YPCDN to create a platform to empower and energize students and young professionals to work together and find relevant solutions to local and global NCD issues provide a clear avenue for engagement. The experience in Kenya highlights how such a network can empower the youth to have a substantive impact on the prevention and mitigation of NCDs in their local context. We note this is a model that may have applications in many regions of the world. Via research, education, advocacy, creating awareness, medical camps or networking, young people and emerging leaders have massive potential and should be encouraged and supported to take up active roles in the fight against NCDs.
